# Prospective evaluation of a decision support system providing advice on ventilator settings of: inspiratory oxygen, delivered pressure or volume and frequency

**DOI:** 10.1186/2197-425X-3-S1-A281

**Published:** 2015-10-01

**Authors:** SE Rees, N Dey, DC Lodahl, A Ciubotariu, J Pilypaite, RR Winding, DS Karbing

**Affiliations:** Aalborg University, Health Science and Technology, Aalborg, Denmark; Herning Hospital, Herning, Denmark; Holsterbro Hospital, Holsterbro, Denmark; Hjørring Hospital, Hjørring, Denmark; Aalborg University Hospital, Aalborg, Denmark

## Introduction

Selecting, and adapting, appropriate settings of mechanical ventilation can be seen as difficult and time consuming. Clinical guidelines have been shown to be helpful, but do not provide advice that is tuned to the individual patient. The Beacon Caresystem (Mermaid Care, Denmark) is a commercial version of a previously published [1] physiological-model based system for advising on mechanical ventilation. Mathematical models are tuned to patient specific measurements, and as such advice is for the individual patient. Beacon 3 provides advice for control and support modes of ventilation, with advice given on: inspiratory oxygen; inspiratory pressure or tidal volume; and, in control modes, respiratory frequency.

## Objectives

This study investigates the changes in oxygenation, ventilation and acid-base status in patients where the system is used for a maximum of 8 hours.

## Methods

Patients have been recruited from non-cardiac intensive care units at four hospitals in Denmark. Informed consent and ethical approval was obtained in all cases. A mix of patients have been recruited ranging from those classified as mild to moderate ARDS, to those during weaning. The study plans inclusion of 60 patients, with 25 included to date. All p-values are calculated using paired t-test.

## Results

Fifteen patients were in pressure support and 10 in volume control modes. Advice was provided an average of 4 times for each patient. Average values of ventilation, oxygenation and acid-base status across all patients at the start and end of using the system were as follows. Inspired oxygen did not change with a small, insignificant, reduction in FI02 from 38.2 ± 7.0% to 36.4 ± 8.6 (p = 0.18). A small reduction in arterial oxygen partial pressure (from 12.1 ± 2.4 kPa to 10.9 ± 2.2 kPa, p = 0.06) and saturation (97.2 ± 1.5 to 95.9 ± 1.4, p < 0.01) was seen. Ventilation was on average reduced, with tidal volumes falling from 586 ± 201 ml to 536 ± 166 ml (p = 0.06), and little change in average respiratory frequency (from 20.0 ± 9.3 breaths/min to 20.7 ± 8.5 breaths/min, p > 0.5). The reduction in ventilation induced a decrease in average plateau pressure (from 20.5 ± 6.1 cmH2O to 17.3 ± 5.2 cmH2O, p < 0.01), and modified acid base status only slightly (pH from 7.428 ± 0.044 to 7.402 ± 0.041, p < 0.05; PaC02 from 5.3 ± 1.0 kPa to 5.7 ± 0.6 kPa, p < 0.05; FETC02 from 4.5 ± 0.8 % to 4.8 ± 0.7%, p= 0.06).

## Conclusions

These initial results indicate that the Beacon Caresystem provides advice which is rational and tailored to the individual patient's physiology, lowering ventilation without adversely affecting acid-base status or oxygenation.

## Grant Acknowledgment

SER and DSK are minor shareholders and perform consultancy work for Mermaid Care.
